# Comparative evaluation of antimicrobial properties of silver nanoparticles and chlorhexidine mouthwashes on the colonization of microflora and oral health during orthodontic treatment: a double-blind randomized controlled trial

**DOI:** 10.1590/2177-6709.30.1.e2524112.oar

**Published:** 2025-04-07

**Authors:** Pradeep RAGHAV, Amit Kumar KHERA, Shrestha BISHT

**Affiliations:** 1 Swami Vivekanand Subharti University, Subharti Dental College, Department of Orthodontics (Uttar Pradesh, India).

**Keywords:** Silver nanoparticles, Chlorhexidine, Mouthwash, Orthodontic appliances, Dental caries, Oral health, Nanopartículas de prata, Clorexidina, Enxaguante bucal, Aparelhos ortodônticos, Cárie dentária, Saúde bucal

## Abstract

**Introduction:**

Fixed orthodontic appliances facilitate microbial adhesion and plaque accumulation. Although chlorhexidine has proven to be the most effective antiplaque and antigingivitis agent, it has several side effects.

**Objective:**

The present study aims to evaluate and compare the effectiveness of silver nanoparticles (SNP) and Chlorhexidine (CHX) mouthwash on reducing the microbial count and improving oral health during fixed orthodontic treatment.

**Trial design:**

4-arm parallel, placebo-controlled, double-blind randomized clinical trial with an allocation ratio of 1:1:1:1.

**Methods:**

Microbial count of *Streptococcus spp.*, Plaque Index, Gingival Index, Gingival Bleeding Index and Pocket Probing Depth were evaluated in 80 orthodontic patients at T0 (Pretreatment), T1 (four weeks after bonding) and T2 (four weeks after mouthwash prescription). Then patients were randomized into four groups: G1) NanOlife (containing SNP), G2) Chlorhex (containing 0.2% chlorhexidine), G3) Placebo, and G4) negative control (no mouthwash prescription) (n=20 each group). The effects of orthodontic treatment and mouthwashes were analyzed statistically.

**Results:**

The microbial count and all the four indices increased between T0 and T1 (P<0.05). They decreased below the baseline levels in Gp2, and back to baseline levels in Gp1 at T2 (P<0.05), except Gingival Bleeding index and Pocket Probing Depth. Both mouthwashes showed significantly improved therapeutic effects compared to placebo and negative control.

**Conclusions:**

Although SNP mouthwash was effective in the reduction of all the assessed parameters, it could not decrease them to the baseline levels, suggesting its limited efficacy. CHX mouthwash proved to be extremely effective by decreasing the microbial count, plaque index and gingival index below the baseline level.

## RESUMO

**Introdução:**

Aparelhos ortodônticos fixos facilitam a adesão microbiana e o acúmulo de placa bacteriana. Embora a clorexidina tenha provado ser o agente antiplaca e antigengivite mais eficaz, ela apresenta vários efeitos colaterais.

**Objetivo:**

O presente estudo teve como objetivo avaliar e comparar a eficácia dos enxaguantes bucais com nanopartículas de prata (NPP) e com clorexidina (CHX) na redução da contagem microbiana e na melhoria da saúde bucal durante o tratamento ortodôntico fixo.

**Desenho do estudo:**

ensaio clínico randomizado duplo-cego, paralelo, controlado por placebo, de quatro braços, com proporção de alocação de 1:1:1:1.

**Métodos:**

Foram avaliados: a Contagem Microbiana de *Streptococcus spp.*, o Índice de Placa, Índice Gengival, Índice de Sangramento Gengival e a Profundidade de Sondagem da Bolsa, em 80 pacientes ortodônticos em T0 (Pré-tratamento), T1 (quatro semanas após a colagem) e T2 (quatro semanas após a prescrição dos enxaguantes bucais). Em seguida, os pacientes foram randomizados em quatro grupos: G1) NanOlife (contendo NPP), G2) Chlorhex (contendo clorexidina 0,2%), G3) Placebo, e G4) controle negativo (sem prescrição de enxaguante bucal) (n=20 cada grupo). Os efeitos do tratamento ortodôntico e dos enxaguantes bucais foram analisados estatisticamente.

**Resultados:**

A contagem microbiana e todos os quatro índices aumentaram entre T0 e T1 (P<0,05). Eles diminuíram abaixo dos níveis basais no Gp2 e voltaram aos níveis basais no Gp1 em T2 (P<0,05), exceto pelo índice de sangramento gengival e a profundidade de sondagem da bolsa. Ambos os enxaguantes bucais mostraram efeitos terapêuticos significativamente melhores em comparação com o placebo e o controle negativo.

**Conclusões:**

Embora o enxaguante bucal com NPP tenha sido eficaz na redução de todos os parâmetros avaliados, não conseguiu reduzi-los aos níveis basais, sugerindo uma eficácia limitada. O enxaguante bucal com CHX provou ser extremamente eficaz em diminuir a contagem microbiana, o índice de placa e o índice gengival abaixo do nível basal.

## INTRODUCTION

The complex design and surface characteristics of orthodontic brackets, bands, and other attachments alter the ecological environment of the oral cavity, facilitating microbial adhesion and plaque accumulation.^1^ Furthermore, they jeopardize oral hygiene maintenance by interfering with natural self-cleansing. This accumulated plaque induces gingivitis and periodontitis, which might interfere with the orthodontic outcome by inhibiting the connective tissue and bone remodeling.^2^ It also results in the development of dental caries and white spot lesions, further aggravating the problems.^3^ Consequently, strict oral hygiene maintenance practices have become imperative for orthodontic patients.

Antimicrobial agents are prescribed by most orthodontists as an adjunct, as it has been proven that combined utilization of mechanical and chemical oral hygiene is more advantageous.^4^ Amongst these, chlorhexidine gluconate has been extensively studied and has proven to be the most effective antiplaque and antigingivitis agent at present.^5^ Despite this, it has several detrimental local side effects like reversible brownish discolouration of teeth and oral mucosa, taste perturbation, hypersensitivity, parotid duct stenosis^6^ in severe cases, and a detrimental effect on the healthy oral commensals.^7^ These side effects add to the growing body of evidence, creating the need for the development of alternative antiplaque agents.

Silver and its derivatives are amongst the oldest antimicrobial agents used in traditional medicine.^8^ Currently, silver nanoparticles (SNP) are used widely as a health care adjunct and have attracted extensive research in the fields of dentistry and orthodontics. SNP release silver ions that disrupt the integrity of bacterial membranes, cling to bacterial DNA and proteins, and modulate the phosphotyrosine profile of putative bacterial peptides, affecting bacterial signal transduction and thus inhibiting their growth.^9^ Furthermore, since SNP present a greater surface-to-volume ratio, they provide a considerably larger surface area for antimicrobial activity. Previous studies were mainly conducted by incorporating the SNP in the orthodontic adhesives, to evaluate its antimicrobial effect. A recent systematic review confirmed that the incorporation of SNP in orthodontic adhesives enhanced its antibacterial efficacy.^10^ Retnaningrum et al.^11^ also conducted a study to investigate the effect of silver nanoparticles prepared from green nanotechnology added into the orthodontic adhesive on bracket’s tensile bond strength (TBS), and the authors concluded that SNP above 3% concentration decrease the TBS, but were still in the recommended adequate bond strength range. 

However, based on the available orthodontic literature, it appears that most of previous researches were done *in vitro* and incorporating the SNP in orthodontic adhesives, which may compromise its bond strength. So, to overcome this issue, the present study used a commercially available mouthwash containing SNP (NanOlife, Dhanvantri Nano Ayushodi Pvt. Ltd). The mouthwash is alcohol-free and contains 100% SNP prepared by innovative Green Nanotechnology.

 The clinical rationale for using SNP mouthwash was its proven antibacterial property and the requirement of a mouthwash having fewer or negligible side effects.

To the best of our knowledge, at the time the current trial was designed, none of the available literature has evaluated the effect of SNP-incorporated mouthwash on the microbial count and oral health indices during orthodontic treatment. Moreover, the comparative evaluation with the gold standard, chlorhexidine mouthwash (CHX), has also not been performed. Thus, the current trial was conducted to evaluate and compare the effectiveness of SNP mouthwash and CHX mouthwash on reducing the microbial count and improving oral health during fixed orthodontic treatment.

The null hypothesis was that there was no difference in the microbial count and oral health indices before and after the use of SNP and CHX mouthwashs.

Specific hypothesis: The primary objective was to evaluate and compare the effectiveness of SNP mouthwash and CHX mouthwash on the colonization of microorganisms during fixed orthodontic treatment; and the secondary objective was to evaluate and compare the effectiveness of SNP mouthwash and CHX mouthwash on gingival health during fixed orthodontic treatment.

## MATERIAL AND METHODS

Trial design: This double-blind, placebo-controlled randomized controlled clinical trial was conducted on 80 fixed orthodontic patients (n=20 each group). The trial was a 4-arm parallel, placebo-controlled randomized clinical trial, with an allocation ratio of 1:1:1:1.

Registration, ethical considerations, and potential harms The study protocol was pre-designed before being carried out as per the pilot study, and remained unchanged during the study. The trial was registered at the Institutional Ethics Committee (IEC) with the registration number SDC/IEC/2020/669(12). The study was also monitored and approved by the IEC, and prior consent of the participating patients was taken (if the participating patient was a minor, then the informed consent of the guardian/parent was taken). The study followed the declaration of Helsinki guidelines (World Medical Association 2013), and the presentation of the report is according to the CONSORT guidelines^12^.

PARTICIPANTS, ELIGIBILITY CRITERIA, AND SETTING

The patients were selected from O.P.D. and the ongoing patients in our department during 2020-2021. The subjects were systematically recruited according to the eligibility criteria (Table 1). 

**Table 1:** Inclusion and exclusion criteria.

	INCLUSION CRITERIA	EXCLUSION CRITERIA
1.	Subjects who needed to undergo fixed orthodontic treatment within the age group of 15-40 year	History of previous periodontal treatments and/or active periodontal diseases and active caries
2.	Absence of severe dental plaque (plaque index ≤ 20% measured by O’Leary index using plaque disclosing agent) / severe gingival inflammation / severe periodontal condition	Long term use of antibiotics, phenytoin, cyclosporine, systemic corticosteroids, calcium channel blockers and anti-inflammatory drugs
3.	Absence of any systemic disease	History of smoking, pregnancy and lactation, dental treatment - crown and bridge/ restorations near the gingival margins
4.	Patients should not be using any mouthwash for at least 1 month before the initiation of the study	History of parafunctional and deleterious habits (tobacco chewing), mouthbreathing or any other problems that could affect oral microbial flora

### INTERVENTION

The samples were randomly divided into three experimental groups and one negative control group. All four groups had 20 participants each (n=80).

» Group 1 - silver nanoparticles mouthwash. (NanOLife, Dhanvantri Nano Ayushodi Pvt. Ltd.).
» Group 2 - 0.2% chlorhexidine mouthwash (Dr Reddy’s Chlohex).
» Group 3 - placebo mouthwash (250 mL distilled water with artificial mint flavor).
» Group 4 - negative control group, no mouthwash was prescribed.

## UNIFORM TREATMENT PROTOCOL

Before the beginning of orthodontic treatment, a periodontist performed oral prophylaxis for all the patients. Immediately before the beginning of the orthodontic treatment, oral health indices and plaque samples (detailed below) were recorded by the same periodontist and the principal investigating orthodontist, respectively. Afterwards, the fixed orthodontic treatment was started with the following protocol. Preadjusted Edgewise appliance with 0.018 × 0.025-in MBT (Ormco Corporation, California) prescription was used. The trial was conducted during initial alignment and leveling stage in which nickel-titanium (American Orthodontics) archwires were used. All the patients received identical and standardized written and oral instructions for hygiene maintenance, including the advice to brush their teeth at least two times daily and dietary instructions were also given, such as: eating three times/day, avoid sticky, gummy, chewy, or very hard food. They were prescribed a single type and brand of toothpaste (Colgate Strong Teeth) and toothbrush (STIM Orthobrush), and were taught Charter’s brushing technique. Patients were motivated to maintain good oral hygiene by reinforcement of the former instructions at every appointment. 

## MOUTHWASH APPLICATION PROTOCOL

Patients were provided with oral and written instructions by the orthodontist to use each mouthwash, according to its manufacturer’s recommended regime, without disclosing the type of mouthwash to the patients.

i) Group 1: Patients were instructed to use silver nanoparticles mouthwash twice a day, in doses of 15 mL, swished for 30 seconds. Patients were asked to avoid eating or drinking for about 30 minutes after using the mouthwash.
ii) Group 2: Patients were instructed to use chlorhexidine mouthwash similarly and were advised to give a gap of 30 minutes between the brushing and the mouthwash, to avoid the interaction between the anionic toothpaste and cationic mouthwash.
iii) Group 3: The same regime was used in the placebo group.
iv) Group 4: Strict instructions were given not to use any type of mouthwash.

The washout period between bonding of fixed orthodontic appliance and start of mouth wash trial was kept four weeks and, after bonding of fixed orthodontic appliance, participants were strictly instructed not to use any type of mouthwash for the next four weeks. This improved the internal effectiveness of the trial.

Sampling was performed at three intervals: T0 - before bonding of the fixed orthodontic appliance, T1 - four weeks after bonding and T2 - four weeks following the mouthwash treatment.

### OUTCOMES

#### Primary outcome

To evaluate and compare the effectiveness of silver nanoparticles mouthwash and chlorhexidine mouthwash on the colonization of microorganisms during fixed orthodontic treatment.

Plaque samples were collected for the assessment of microbial count at T0, T1 and T2. The participants were instructed to use a disclosing agent as per the manufacturer’s instructions. The supragingival plaque was collected with a sterile #23 explorer from the cervical thirds of the buccal surfaces of four index teeth: #12, #24, #32 and #44. It was moved only once over the tooth surfaces, for a uniform sample collection. Plaque samples collected at each evaluation were transferred to a test tube containing 5mL of Robertson’s Cooked Meat Broth. After immediate transfer to the laboratory, samples were homogenized and inoculated onto the selective agar medium, for the *Streptococcus* group. Samples were tested for the presence of *Streptococcus spp.* as it is the most common cause for tooth demineralization. After 48 hours of incubation at 37°C with 5-10% CO_2_, the numbers of *Streptococcus spp.* colonies were determined.

For the microbial count, countable dilution giving up to 50-500 colonies was done. The countable colonies were converted into CFU/mL by multiplying to its dilution factor. Each test was performed in triplicate and finally, the mean value of CFU was reported. 

Presumptive identification of colonies on selective media was based on colonial morphology, counted in the digital colony counter, and was confirmed by Vitek^®^ 2 GP ID cards. The VITEK 2 gram-positive (GP) identification cards (bioMérieux) were used as it is a highly automated identification system, enabling more rapid, accurate and cost-effective identification of medically relevant gram-positive cocci.^13^ Funke and Funke-Kissling^14^ reported the correct identification of >94% of the isolates to the species level without any additional confirmatory tests. 

#### Secondary outcome

To evaluate and compare the effectiveness of silver nanoparticles mouthwash and chlorhexidine mouthwash on gingival health during fixed orthodontic treatment.

All the gingival examinations were carried out by the sole periodontist involved in this study, to ensure reliability. The examined gingival indices were Sillness and Löe plaque index (PI), Löe and Silness gingival index (GI), Ainamo and Bay gingival bleeding index (GBI), and pocket probing depth (PPD). The PI was examined on all teeth after using a disclosing agent, while GI, GBI, and PPD were assessed using a UNC-15 probe. The gingival examination was carried out three times (T0, T1 and T2). The data was tabulated and sent for statistical analysis.

### SAMPLE SIZE CALCULATION

The sample was calculated to minimum power of 95% and an α error of 0.05 and Cohen’s effect size of 0.89 (large effect size). The sample size was determined using the G-Power software. The effect size was estimated by using the data obtained from a previous study conducted by Sobouti et al.^15^ The calculations yielded a required sample of 19 per group for a two-tailed study. Since the present study had four groups, the total sample size was determined to be 80.

### RANDOMIZATION AND BLINDING

Randomization and sequence generation was performed by Random Allocation Software. The concealment of treatment modality was done in sequentially numbered, opaque and sealed envelopes, which were shuffled by the investigator. Thereafter, each subject was given an envelope and was allotted to one of the four study groups. The coded list of randomized patients was concealed within a sealed envelope to which only the data analyst had access. The test mouthwashes and the placebo were given to the participants in identical containers. An independent investigator, who was blinded, poured the mouthwashes from their commercial containers into those containers, ensuring double-blinding. Neither the operator nor the patients were aware of the type of mouthwashes. 

### STATISTICAL DATA ANALYSIS

The data on continuous variable was shown as mean and standard deviation (SD) across the four study groups. A test of normality and the equality of variances in the samples were conducted. A two-way repeated measures analysis of variance (ANOVA) was used for the evaluation of the effects of time and types of mouthwash. Post-hoc LSD test was performed for multiple group comparisons using a commercially available program (SPSS 12 for windows, SPSS Inc., Chicago, IL, USA) with the level of significance at 0.05. 

## RESULTS

### PARTICIPANT FLOW

**Figure 1:**
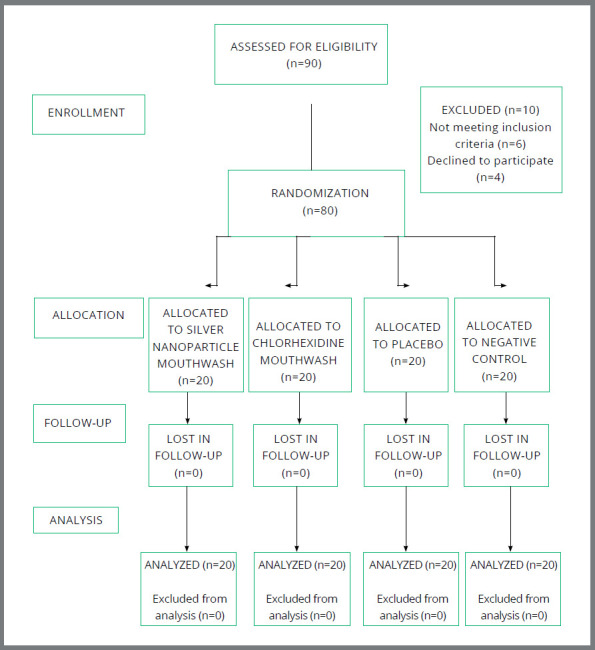
CONSORT flow diagram.

The total number of patients was 80 (38 males and 42 females) with a mean age of 20.02 ± 3.91 (15-40) years, distributed into four groups of 20 patients. A flow chart with participant flow and dropouts is presented in Figure 1. The clinical examination was conducted from August 2021 to November 2022. All results are shown in tabular as well as graphical format, to visualize the statistically significant difference more clearly.

### BASELINE DATA

Table 2 shows the baseline clinical characteristics for each group. The baseline variables of all groups were compared, and no statistically significant difference was found between them.

**Table 2:** Baseline clinical characteristics.

	Group 1 (n=20)	Group 2 (n=20)	Group 3 (n=20)	Group 4 (n=20)	P-value
Age (years)	18.8 ± 3.51	19.85 ± 3.45	21.4 ± 4.44	20.05 ± 4.09	0.259^NS^
Sex (female)	45%	40%	50%	45%	0.568^NS^
mean bacterial count (CFU/mL) 10^4^	1.97 ± 0.66	2.26 ± 0.78	1.73 ± 0.45	2.18 ± 0.81	0.079^NS^
mean plaque index	1.23 ± 0.33	1.36 ± 0.33	1.07 ± 0.18	1.19 ± 0.36	0.138^NS^
mean gingival index	1.29 ± 0.37	1.38 ± 0.32	1.09 ± 0.20	1.21 ± 0.39	0.122^NS^
mean gingival bleeding index	53.65 ± 10.36	57.10 ± 9.45	49.20 ± 7.46	49.50 ± 10.11	0.105^NS^
mean pocket probing depth	1.64 ± 0.31	1.76 ± 0.51	1.49 ± 0.17	1.60 ± 0.38	0.169^NS^

### NUMBER ANALYZED FOR PRIMARY AND SECONDARY OUTCOMES AND SUBGROUP ANALYSIS

There was no dropout during the trial. So, at the end of this trial, 20 patients in each group were analyzed for primary and secondary outcomes.

### OUTCOMES AND ESTIMATION

#### ASSESSMENT OF MICROBIAL COUNT AND GINGIVAL HEALTH

Table 3 shows mean and standard deviation for Bacterial count (CFU), Plaque index (PI), Gingival index (GI), Gingival bleeding index (GBI) and Pocket probing depth (PPD) for all four groups at different time intervals.

**Table 3:** Mean and standard deviation for Bacterial count (CFU), Plaque index (PI), Gingival index (GI), Gingival bleeding index (GBI) and Pocket probing depth (PPD) for all four groups at different time intervals.

Group	Time interval	CFU	PI	GI	GBI	PPD
SNP	Baseline	1.97 (0.66)	1.22 (0.32)	1.29 (0.37)	53.65 (10.3)	1.64 (0.30)
	Mid	2.75 (0.86)	1.79 (0.32)	1.81 (0.45)	64.25 (8.9)	1.69 (0.32)
	Post	1.88 (0.76)	1.36 (0.32)	1.4 (0.48)	53.8 (9.3)	1.60 (0.31)
CHX	Baseline	2.25 (0.77)	1.35 (0.32)	1.38 (0.32)	57.1 (9.4)	1.75 (0.51)
	Mid	3.19 (0.89)	2.02 (0.37)	2.02 (0.37)	66.9 (7.9)	1.82 (0.50)
	Post	1.68 (0.57)	1.14 (0.18)	1.14 (0.18)	52.65 (8.4)	1.64 (0.45)
Placebo	Baseline	1.73 (0.44)	1.07 (0.18)	1.09 (0.19)	49.2 (7.4)	1.49 (0.17)
	Mid	2.63 (0.90)	1.67 (0.16)	1.79 (0.34)	62.7 (8.2)	1.6 (0.17)
	Post	2.56 (0.78)	1.61 (0.33)	1.78 (0.37)	62.3 (7.1)	1.58 (0.20)
Control	Baseline	2.18 (0.81)	1.19 (0.36)	1.21 (0.38)	49.5 (10.1)	1.60 (0.38)
	Mid	2.99 (1.1)	1.77 (0.36)	1.96 (0.52)	66.4 (11.5)	1.66 (0.33)
	Post	3.41 (1.1)	2.14 (0.39)	2.13 (0.49)	71.6 (8.6)	1.74 (0.36)

A two-way RMANOVA (Table 4) shows the results of assessing the effect of the interaction of time and different treatment groups on bacterial count (CFU), PI, GI, GBI and PPD. Overall, different treatment groups (p=0.028) and different time intervals (p<0.001) had a significant influence on the bacterial count, plaque index and gingival index, but did not have a significant influence on the gingival bleeding index (p=0.088) and pocket probing depth (p=0.43). Similarly, the effect of the interaction of time and different treatment groups had a significant influence on the bacterial count, plaque index and gingival index, gingival bleeding index and pocket probing depth (p<0.001).

**Table 4:** Assessment of the effect of the interaction of time and different treatment groups on Bacterial count (CFU), Plaque index (PI), Gingival index (GI), Gingival bleeding index (GBI) and Pocket probing depth (PPD).

Source	CFU		PI		GI		GBI		PPD	
	F	p-value	F	p-value	F	p-value	F	p-value	F	p-value
Treatment	3.261	0.028*	4.956	0.004*	3.026	0.037*	2.291	0.088	0.935	0.43
Time	689.988	<0.001*	146.129	<0.001*	107.767	<0.001*	49.439	<0.001*	24.55	<0.001*
Treatment* Time	29.473	<0.001*	54.54	<0.001*	28.143	<0.001*	21.1	<0.001*	19.609	<0.001*

Two-way repeated measures ANOVA test; * indicates a significant difference at p≤0.05.

Table 5 and Figures 2A-[Fig f3] [Fig f3b] [Fig f4] [Fig f4b] [Fig f5] [Fig f5b] 6A shows the intergroup comparisons of the mean bacterial count, PI, GI, GBI and PPD respectively, using two way ANOVA with LSD post-hoc test for multiple group comparisons. No statistically significant difference was found between the two tested mouthwashes in reducing the microbial count and improving the oral health indices (p>0.05). However, a significantly higher mean bacterial count, PI, GI, GBI and PPD was found in placebo group, as compared to SNP and CHX groups, but the difference was not statistically significant. Mean bacterial count, PI, GI were statistically significantly higher in control group, as compared to all other three groups, but there was no statistically significant (p>0.05) difference in GBI and PPD among all four groups.

**Table 5:** Intergroup comparisons of Bacterial count (CFU), Plaque index (PI), Gingival index (GI), Gingival bleeding index (GBI) and Pocket probing depth (PPD).

Pairwise comparisons		CFU	PI	GI	GBI	PPD
SNP	CHX	0.414	0.858	0.697	0.667	0.444
	Placebo	0.655	0.627	0.926	0.914	0.205
	Control	0.019*	0.036*	0.023*	0.080	0.820
CHX	Placebo	0.729	0.601	0.702	0.694	0.106
	Control	0.024*	0.028*	0.006*	0.117	0.653
Placebo	Control	0.014*	0.035*	0.003*	0.039*	0.215

P-value by *post-hoc* LSD test. *Statistically significant (P<0.05).

**Figure 2A:**
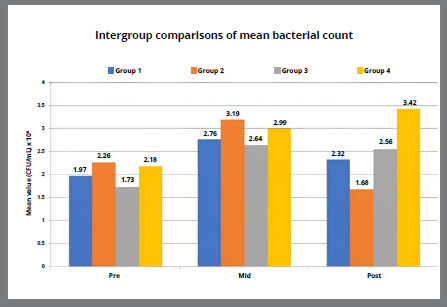
Intergroup comparison of mean bacterial count among groups.

**Figure 2B:**
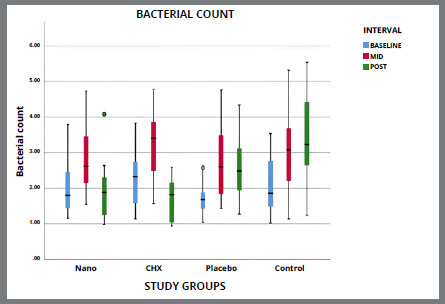
Box plot graph showing the mean bacterial count among different groups at different time intervals.

**Figure 3A: f3:**
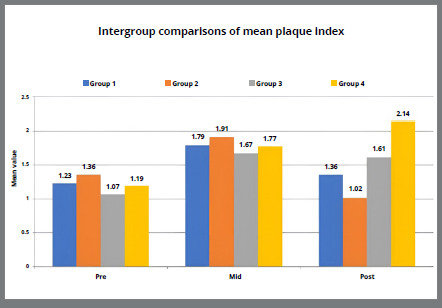
Intergroup comparison of plaque index among groups.

**Figure 3B: f3b:**
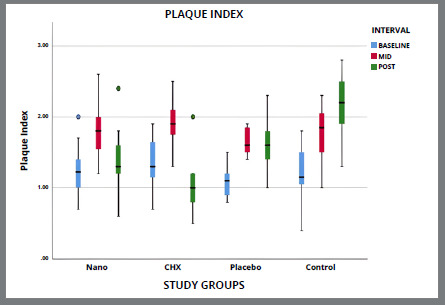
Box plot graph showing the mean plaque index among different groups at different time intervals.

**Figure 4A: f4:**
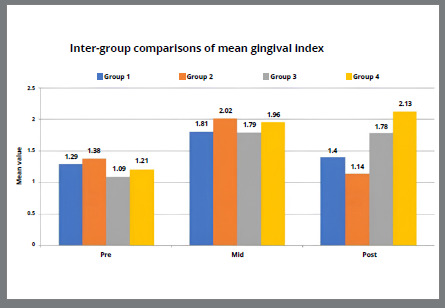
Intergroup comparison of gingival index among groups.

**Figure 4B: f4b:**
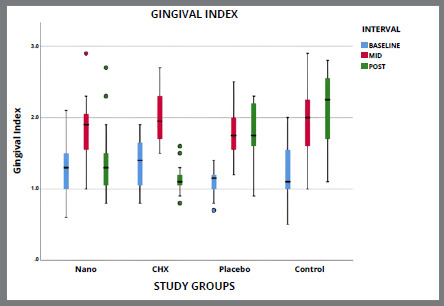
Box plot graph showing the mean gingival index among different groups at different time intervals.

**Figure 5A: f5:**
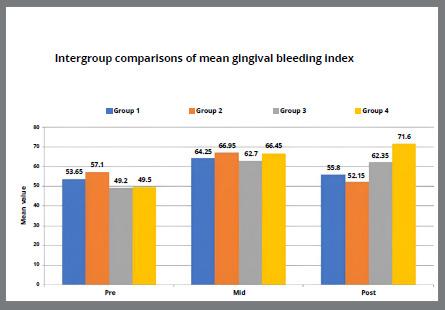
Intergroup comparison of gingival bleeding index among groups.

**Figure 5B: f5b:**
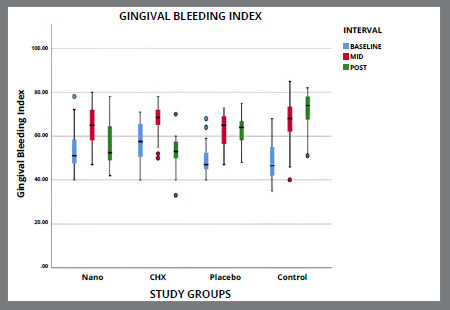
Box plot graph showing the mean gingival bleeding index among different groups at different time intervals.

**Figure 6A: f6:**
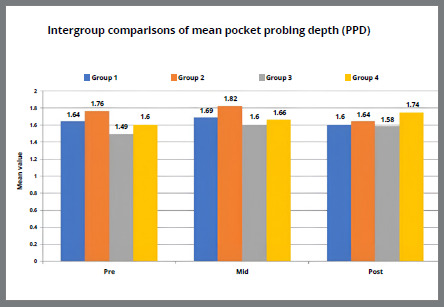
Intergroup comparison of gingival pocket probing depth among groups.

**Figure 6B:**
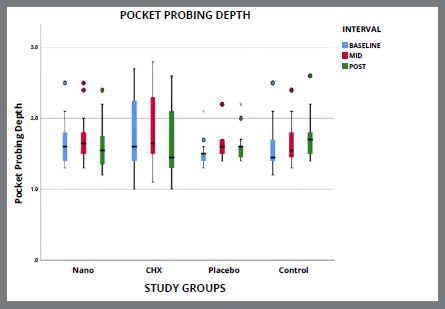
Box plot graph showing the mean pocket probing depth among different groups at different time intervals.

Figures 2B-[Fig f3] [Fig f3b] [Fig f4] [Fig f4b] [Fig f5] [Fig f5b] [Fig f6] 6B shows the mean changes in bacterial count, PI, GI, GBI and PPD at different time intervals between the all four groups. The pairwise comparison at different time periods showed a statistically significant difference for each variable in all the groups, as shown in Table 6.

**Table 6:** Pairwise comparisons of Bacterial count (CFU), Plaque index (PI), Gingival index (GI), Gingival bleeding index (GBI) and Pocket probing depth (PPD).

Pairwise Comparisons		CFU	PI	GI	GBI	PPD
Baseline	Mid	<0.001*	<0.001*	<0.001*	<0.001*	<0.001*
	Post	<0.001*	<0.001*	<0.001*	<0.001*	0.430
Mid	Post	<0.001*	<0.001*	<0.001*	<0.001*	<0.001*

P-value by *post-hoc* LSD test. *Statistically significant (P<0.05).

In all four groups, significant differences were observed between baseline and mid-treatment (p<0.001), baseline and post-treatment (p<0.001) as well as between mid and post-treatment (p<0.001). Rise in bacterial count, PI, GI, GBI and PPD was seen from baseline to mid-treatment, which was statistically significant. The decrease in bacterial count, PI, GI, GBI and PPD from mid to post-treatment was also significant in all three groups other than control group. In control group, there was significant rise in value of all variables from mid to post-treatment.

### HARMS

No harm was detected or reported by the participants during the intervention period of one month. 

## DISCUSSION

### THE MAIN FINDING IN THE CONTEXT OF EXISTING EVIDENCE

Placement of orthodontic appliances makes oral hygiene difficult, leading to an increase in plaque accumulation, enamel demineralization and gingival inflammation ^16^ ^,^ ^17^. Antimicrobial agents are prescribed by most orthodontists as an adjunct, as combined utilization of mechanical and chemical oral hygiene has proven to be more advantageous.^4^ Although many studies have evaluated the efficacy of the available antimicrobials, none has compared the efficacy of the gold standard antimicrobial chlorhexidine (CHX) ant the silver nanoparticles (SNP) mouthwash in reducing the microbial count and improving the gingival health during orthodontic treatment. Thus, the present study was conducted to evaluate and compare the effectiveness of silver nanoparticles mouthwash and chlorhexidine mouthwash on the microbial count and gingival health during fixed orthodontic treatment. 

Among the bacteria present in the oral cavity, *Streptococcus spp*. was selected because they are the principal cariogenic bacteria, and a significant increase in their quantity is found after the placement of orthodontic appliances.^18^ In this study, the effect of both mouthwashes was studied for one month during fixed orthodontic treatment. This was done for two reasons: first, because of the uncertainty of the long-term effects of SNPs mouthwash and the proven side-effects like staining and altered taste caused by CHX mouthwash; and second, because the general plaque index has been shown to improve as the treatment progresses due to a better understanding of the need to maintain oral health.^19^ ^,^ ^20^

A significant increase was found in the mean bacterial count and all the indices after the bonding of fixed appliance after one month (at mid-treatment T1) before the use of mouthwashes, compared to pre-treatment (T0) for all four groups. Our findings in terms of the effect of orthodontic treatment on the worsening of oral health due to increased microbial count were consistent with other studies^18^ ^,^ ^21^ stating that patients undergoing orthodontic treatment show a continuing increase of *Streptococcus spp.* levels at diverse degrees of significance, especially within the initial days of orthodontic treatment.

### EFFECT OF MOUTHWASHES ON MICROBIAL COUNT

Chlorhexidine has long-spectrum antibacterial activity and inhibits plaque formation, as well as reduce gingivitis. On the other hand, silver nanoparticles are also well known for their antimicrobial efficacy.

In the present study, both SNP and CHX groups also showed a significant decrease in colony count after one month using mouthwash (T2, post-treatment) from mid-treatment (T1), but in CHX group mean bacterial count decrease to -24.40%, as compared to SNP group, in which bacterial count reduced only +28.48%, indicating that both mouthwashes were effective, but CHX was more effective, as compared to SNP, because the bacterial count decreased below the baseline level.

The findings of the present study regarding the comparative effectiveness of SNP and CHX mouthwashes on the microbial counts are in accordance with the recently conducted study by Fakhri et al,^22^ which also compared the antibacterial efficacy of AgNPs stabilized with chitosan with 0.2%chlorhexidine against*S. mutans,* and found no significant difference.

The results of this trial showed the efficacy of CHX mouthwash, in line of other previous studies^20^ ^,^ ^23^ ^,^ ^24^. They all reported a significant decrease in *Streptococcus* count after the administration of CHX for 1, 2 and 3 weeks, respectively. According to Enita et al,^25^ the *S. mutans* count decreased from 65% (baseline) to 25% after two weeks of CHX mouthwash usage.

The current study also affirmed the efficacy of SNP mouthwash in terms of reduction in the microbial count. This finding was in line with other previous studies^26^ ^-^ ^29^ reporting the efficacy of SNP-incorporated dental materials. Although there have been researches on SNP-incorporated dental materials including mouthwashes, none of them has investigated the impact on the microbial count and oral health. Ali et al.^26^ reported a significant decrease in the mean with spot lesions in the SNP group, compared to CHX and fluoride groups, at 90 and 180 days of follow-up. In an *in-vitro* trial, Choi et al.^27^ reported that nanosilver fluoride sustained release orthodontic elastomers provides antimicrobial and antibiofilm effects against*S. mutans*. Another in-vitro studies^28^ ^,^ ^29^ reported that the SNP-containing antibacterial adhesive could inhibit the growth of *S. mutans* and*L. acidophilus*. Yassaei et al.^30^ evaluated the long-term effect of composites containing silver and titanium nanoparticles and concluded that 1% silver oxide has only short-term antibacterial effect. Aguiar et al.^31^ investigate the antimicrobial activity and shear bond strength (SBS) using experimental composites with different concentrations of silicon dioxide-coated silver nanoparticles, and reported that there was significantly reduction in*S. mutans*biofilm formation and no statistically significant difference in SBS values. According to Farhadian et al.^32^, the incorporation of SNPs to the acrylic plate provided a significant antibacterial effect against *S. mutans* in clinical conditions, with a mean difference of 40.3 colony counts between the two groups, showing a substantive antimicrobial impact. On the contrary, Kim et al.^33^ reported no significant difference in *S. mutans* count between conventional and silver elastomers. It appears that the antibacterial properties of various nanoparticle materials in clinical conditions differ from those seen *in vitro*. A recent systematic review also concluded that the incorporation of nanoparticles into orthodontic materials improves their antibacterial properties.^34^

In the present trial, distilled water with artificial mint was used as mouthwash in the placebo group, in which microbial count decreased from mid-treatment (T1) to post-treatment (T2), but not to a significant level, indicating that irrigating with water after meals might dilute the carbohydrate remnants, as well as chemical products of bacteria, and might have some hygienic effects. On the contrary, the control group showed a significant increase in the microbial count at T2, compared to T1, affirming the continuing increase of *Streptococcus spp.* levels during orthodontic treatment in the absence of mouthwash usage. 

### EFFECT OF MOUTHWASHES ON ORAL HEALTH

Chlorhexidine and silver nanoparticles mouthwashes, along with antibacterial activity, also have the anti-inflammatory action, by which they reduce the gingival inflammation, leading to improvement in oral health. 

The findings of the present research confirmed that oral health indices differed before and after mouthwash application. Orthodontic treatment without mouthwash application increased all four indices (PI, GI, GBI and PPD) significantly. These results were consistent with previous studies indicating a negative role for orthodontic treatment^15^ ^,^ ^19^ ^,^ ^35^ ^-^ ^39^, while, in contrast, some others failed to find such a correlation.^20^ ^,^ ^40^ The reason for the controversy might be the duration of studies or other methodological heterogeneity, disallowing the contrast to reach an observable level. Additionally, according to Ristic et al.^19^ and Lara-Carrillo et al.,^20^ plaque index might decrease over the course of orthodontic treatment, presumably due to greater patient education and plaque control over time. Another reason for the disagreement might be the employed methodologies, since researchers frequently use only plaque indices. Therefore, the present study used more than one measure of oral health indices, to improve both the comparability and reliability of the results.

The present trial also approves the efficacy of both SNP and CHX mouthwashes in terms of improving all the indices, except gingival bleeding index and pocket probing depth. Although there was improvement in GBI and PPD after the one month use of mouthwashes, the difference was not statistical significant. This might be due to short duration of the study. Both mouthwashes improved the oral health indices similarly, there was no clinically significant difference, but CHX decreased the values below the baseline levels, proving to be more beneficial, as compared to SNP. In Group 3, oral health indices did not worsen from T1 to T2, indicating the role of placebo (distilled water) in maintaining (if not improving) oral health. 

The efficacy of CHX in improving the oral health was in accordance to previous studies^25^ ^,^ ^41^ ^,^ ^42^ ^,^ ^43^. They all reported that chlorhexidine mouthwash improved the gingival conditions in orthodontic patients. 

The effect of SNP on oral health indices during fixed orthodontic treatment was not evaluated in any previous study until date, but the efficacy of silver nanoparticles was evaluated by Kadam et al.^44^ and Hernández Venegas et al.^45^ in chronic periodontitis, and they find that SNP was beneficial in improving the periodontal health.

Even though the benefit of SNP mouthwash in improving oral health is still questionable, the current study supports its role in maintaining oral health after one month of usage in fixed orthodontic treatment. To the best of our knowledge, none of the available literature has evaluated the effectiveness of SNP-containing mouthwash on oral health indices and microbial count during fixed orthodontic treatment. Therefore, future studies should be undertaken *in vivo,* to test the effectiveness as well as side effects of SNP mouthwashes, to drive conclusive results.

### LIMITATIONS OF STUDY

Limitations of this trial could be characterized as: short duration of the study, patients’ compliance could not be ideally controlled, major side effects of CHX -i.e. tooth discolouration and taste perturbation- were neither evaluated nor compared with SNP mouthwash. 

### GENERALISABILITY

Therapeutic effects of both assessed commercial brands were confirmed for both sexes and the tested age groups. Orthodontists may prescribe the use of both test mouthwashes to their patients as coadjuvant means to improve oral health. The mouthwashes would also benefit the general population.

## CONCLUSION

Within the limitations of this study, the following conclusions can be drawn:

1. Fixed orthodontic treatment facilitated increased colonization of microorganisms, which might deteriorate oral and gingival health.
2. Although SNP mouthwash was effective in the reduction of colony counts of *Streptococcus spp.* and improved the oral health indices (PI and GI), it could not decrease them to the baseline levels, suggesting its limited efficacy in terms of microbial count, plaque and gingivitis reduction.
3. CHX mouthwash decreased all the assessed parameters more efficiently, proving to be extremely effective over an intervention period of one month.
4. Both SNP and CHX mouthwashes were effective against *Streptococcus spp.* and hence are a useful adjunct to oral health control methods for orthodontic patients. However, CHX mouth wash was found to be more efficacious than SNP mouthwash.
